# DNA Compaction and Charge Neutralization Regulated by Divalent Ions in very Low pH Solution

**DOI:** 10.3390/polym11020337

**Published:** 2019-02-15

**Authors:** Tianyong Gao, Wei Zhang, Yanwei Wang, Guangcan Yang

**Affiliations:** College of Mathematical, Physics and Electronic Information Engineering, Wenzhou University, Wenzhou 325035, China; wzugty@163.com (T.G.); wzuzww@163.com (W.Z.)

**Keywords:** DNA compaction, charge neutralization, magnesium ions, pH regulation

## Abstract

DNA conformation is strongly dependent on the valence of counterions in solution, and a valence of at least three is needed for DNA compaction. Recently, we directly demonstrated DNA compaction and its regulation, mediated by divalent cations, by lowering the pH of a solution. In the present study, we found that the critical electrophoretic mobility of DNA is promoted to around −1.0 × 10^−4^ cm^2^ V^−1^ s^−1^ to incur DNA compaction or condensation in a tri- and tetravalent counterions solution, corresponding to an about 89% neutralized charge fraction of DNA. This is also valid for DNA compaction by divalent counterions in a low pH solution. It is notable that the critical charge neutralization of DNA for compaction is only about 1% higher than the saturated charge fraction of DNA in a mild divalent ion solution. We also found that DNA compaction by divalent cations at low pH is weakened and even decondensed with an increasing concentration of counterions.

## 1. Introduction

DNA has promising features for use in nanotechnology and is becoming one of the most extensively used molecular building blocks for engineering self-assembling materials [1,2]. In self-assembling materials, DNA molecules are closely packed, suggesting that electrostatic repulsion between negatively charged DNA in the condensed state is balanced by counterion-induced attraction [3]. The condensation of the highly-charged stiff polymer occurs only in presence of multivalent ions, or other functionally similar condensing agents [4]. On the other hand, DNA compaction is closely related to its charge neutralization, and even more, to overcompensation of its electric charge, implying that its net charge switches from negative to positive due to the binding of counterions [5]. The identification of the modes of compaction was possible thanks to the remarkable contribution of the Yoshikawa group, who brought the analysis of DNA compaction to the level of individual molecules [6,7]. They showed three pathways that can be followed by DNA to go from the elongated coil state to the compact state. The first type is an all-or-none compaction process where there is no intermediate state. Instead, there is coexistence between the elongated coil state and the compact state. This process is usually observed when attraction is induced between DNA monomers all along the chain, either by adding small multi-valent counterions or by inducing unfavorable contacts between DNA monomers and the solvent. This is similar to the first-order phase transition between a disordered gas phase (coil state) and a highly condensed solid phase (compact state) [8]. The second mode of compaction is a progressive transition from the elongated coil state to the compact state. This usually occurs when a strong attraction between several consecutive DNA monomers can be induced locally, typically upon complexation with polycations longer than 10 monomers [8]. The two precedent modes of compaction account for a collapse of DNA, resulting from DNA monomer–monomer attraction interactions. The highly packaged structure of DNA inside viruses probably results from a combination of these two modes. The third possible route is an assisted, hierarchical compaction by DNA adsorption and wrapping around nanoscale objects. This is the mode of compaction of DNA into chromatin in eukaryotic cells, and it is observed in vitro when DNA is compacted by cationic nanoparticles [9,10] or dendrimers [11].

The Yoshikawa group revealed the effects of divalent and trivalent cations on the higher-order structure of giant DNA by fluorescence microscopy. It was found that divalent cations, Mg^2+^ and Ca^2+^, inhibit DNA compaction induced by a trivalent cation, spermidine. On the other hand, in the absence of spermidine, divalent cations cause shrinkage of DNA. For the compaction with spermidine, we consider the increase in translational entropy due to the ion-exchange of the intrinsic monovalent cations condensing on a highly charged polyelectrolyte, double-stranded DNA, by the 3+ cations. In contrast, the presence of 2+ cation decreases the gain of entropy contribution by the ion-exchange between monovalent and 3+ ions [12].

DNA condensation and its charge inversion have been experimentally demonstrated by dynamic light scattering (DLS) [13], atomic force microscopy (AFM) [14], and recently developed single molecular techniques such as optical tweezers (OT) [15] and magnetic tweezers (MT) [16]. In general, DNA compaction or condensation occurs in mild pH conditions, when the valence of counterions is larger than 3, while tetravalent or higher cations are able to induce the charge inversion of DNA [17,18]. The Murayama group observed DNA collapse and the occurrence of reentrant transition with a tri-valent cation, spermidine [19]. However, spermidine is not able to lead to DNA charge inversion as described in [20].

In theory, how much of the DNA charge that has to be neutralized to induce its compaction is still controversial. If we approximate DNA as a uniformly charged cylinder and neglect the discreteness of DNA charge and other structural features, then according the Oosaga-Manning theory [12], its fraction of charge neutralization can be simply expressed as

(1) $$\theta =\;1-\;\frac{1}{Z\xi }$$

where *ξ* = $\frac{l_{B}}{b}$, $l_{B}=\frac{q^{2}}{4\pi \varepsilon \varepsilon_{0}k_{B}T}$, *ξ* is charge coefficient of Manning, $l_{B}$ is Bjerrum length, *b* is the *θ* = 1 −$\;\frac{1}{Z\xi }$ average spacing between charges along the chain, −***q*** is the charge of electron, *ε* is the dielectric constant of solution, $\varepsilon_{0}$ is the dielectric constant of vacuum, $k_{B}$ is the Boltzmann constant and *T* is temperature of the solution. For example, the charge fractions of DNA at room temperature in aqueous solution are 76%, 88%, and 92% for mono-, di- and tri-valent counterions, respectively. As DNA is compacted in a solution of trivalent counterions, 92% of the phosphate charge along the DNA backbone to be screened is the upper limit for DNA condensation. Thus, the exact measurement of the critical charge neutralization is helpful in understanding the physical origin of the “like-charge attraction”, and further mechanisms of DNA condensation.

Common mono- and divalent cations such as Na^+^, K^+^, Ca^2+^, and Mg^2+^ (valence <3) are dominant for all DNA and RNA-involved biological processes [21]. In a restricted environment, such as inside a viral capsid, even divalent counterions such as Mg^2+^ can also induce DNA condensation similar to that of trivalent/tetravalent counterions [22]. Synchrotron X-ray diffraction experiments have shown evidence for attraction between lambda-phage DNA in divalent electrolytes when DNA was confined to a two-dimensional cationic surface [23]. Notably, condensation of DNA by divalent Mg^2+^ counterions has never been observed in bulk solution. These studies suggest that any attractive force induced by divalent ions must be weak, which is consistent with the observation that condensation induced by divalent ion of DNA occurs only in reduced dimensions [24].

Recently, we unambiguously demonstrated DNA attraction and its regulation mediated by divalent cations Mg^2+^ and Ca^2+^, by tethering a DNA single chain by lowering the pH of a solution. It has been found that DNA is compacted when the pH of solution containing these divalent counterions is lowered below 5.0 [25]. In the present study, we try to find the critical charge neutralization needed for DNA attraction or compaction. We systematically measured the electrophoretic mobility (EM, μ) of DNA at various ionic conditions and observed the corresponding morphology by atomic force microscopy. We found that the electrophoretic mobility of DNA is promoted to around −1.0 × 10^−4^ cm^2^ V^−1^ s^−1^ to incur DNA compaction or condensation in solution, which corresponds to the charge of DNA that is neutralized by about 89%. The critical electrophoretic mobility of DNA compaction is universal and independent of valence of counterions in solution. We have to point out that the present study is purely in the domain of physical chemistry, and not in the range of physiological conditions. Understanding DNA charge neutralization and compaction at extreme conditions might provide insights for designing and developing molecular structures or devices to perform certain desired functions. On the other hand, intracellular pH regulation is important for controlling the cell cycle and the proliferative capacity of cells, and pH-regulated DNA-based nanomaterials and nanodevices have promising applications for in vivo imaging, clinical diagnostics, and drug delivery.

## 2. Experimental Procedures

### 2.1. Materials

Double-strand λ-phage DNA (48502 bp) was purchased from the New England Biolabs company (Ipswich, MA, USA) and did not go through purification before use. The stoke solution of DNA is 1×TE buffer (10 mM tris-HCl and 1 mM EDTA, pH = 8.0) and the original concentration of DNA is 500 ng μL^−1^. The chemical and biochemical agents (such as magnesium chloride, spermine, spermidine, zwitterionic species glycine, bovine serum albumin (BSA), and hydroxylmethylaminoethane (tris)) were purchased from Sigma-Aldrich and used as received. Phosphate buffer saline (PBS) was used in our sample preparation of MT, containing 10mM phosphate, 140 mM NaCl at pH 8.0. The electrophoretic mobility of DNA at various concentrations of magnesium chloride was measured in a 10 mM tris buffer at different pH values. All solutions were prepared with deionized water with a resistivity of 18.2 MΩ·cm and purified through the Milli-Q system (Millipore Corporation, Burlington, MA, USA). All measurements are repeated at least 3 times to obtain consistent results, and the standard deviation was calculated accordingly.

### 2.2. Methods

#### 2.2.1. Electrophoretic Mobility Measurement

The electrophoretic mobility measurement was carried out by using a DLS device, a Malvern Zetasizer nano ZS90 (Malvern, UK) equipped with its patented M3-PALS technique, in which a He-Ne laser (l = 633 nm) is applied and an avalanche photodiode is used to detect the scattering light. DNA was diluted to a concentration of 1 ng μL^−1^ in 10mM tris buffer by adding magnesium chloride of different concentrations in solutions at various pHs or by adding spermine and spermidine with different concentrations. Before measurement, 5 min are needed for incubation at room temperature. During the process of measurement, sample cell was kept at a constant temperature of 25°. The volume of sample is 1 mL in the folded capillary cell.

#### 2.2.2. AFM Imaging

The AFM was performed in air using a multi-mode AFM with a nanoscope controller (SPM-9600, Shimadzu, Kyoto, Japan) in the tapping-mode. All AFM images were captured with scan speeds of 2 Hz and smoothed manually using off-line analysis software equipped with the microscope. The data collection was 512 × 512 pixels. All manipulations were carried out in 10 mM tris (different pH values) with a final concentration of 1 ng μL^−1^ DNA and the specified concentration of magnesium chloride, spermine, and spermidine. The mixture was deposited onto freshly cleaved mica (1 cm diameter) and incubated for 5 min at room temperature. After incubation, the samples were rinsed with a flow of 20 μL water ten times, and then rapidly blown dried using compressed nitrogen. Balhorn and colleagues have shown that treatment of DNA adsorbed to a mica surface with a solution of multivalent cations produced toroids with lateral dimensions (i.e., diameters), similar to those in solution, but with smaller heights (the axial direction) [26]. However, the condensed DNA is adsorbed on a mica surface and binds to the surface tightly, so the washing of water did not change the DNA morphologies. Thus, AFM images flatten the morphology of DNA condensates, but keep their original compacting structures.

#### 2.2.3. Magnetic Tweezer Tethering

**Figure 1:**
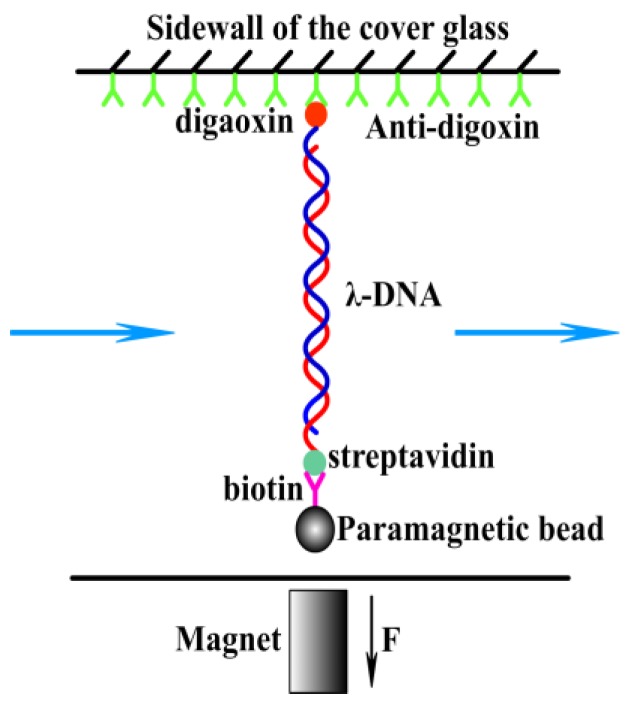
A schematic diagram of magnetic tweezers.

A transverse MT was used to obtain the force spectroscopy of DNA in counterion solution. The detail of setup is as described before [16,27]. In brief, the flow chamber with a polished sidewall was supplied with anti-digoxygenin at first and then was rinsed with PBS containing 5 mg mL^−1^ BSA at pH 8.0. DNA-bead constructs were then flushed into the cell, then a sidewall-DNA-paramagnetic bead structure was formed, as shown in Figure 1. In our MT experiment, PBS buffer was used in the protocol to attach DNA to the sidewall of sample cell, since Na^+^ in PBS would promote the specific binding of biotin and streptavidin or anti-digoxygenin and digoxygenin. After the attachment, the PBS buffer was eluted by tris buffer. The same tris buffer was used in the AFM and DLS experiments. Therefore, the final experiment buffer does not contain monovalent cations. The applied force to the bead is related with Brownian motions of the microsphere and can be calculated accordingly. The DNA extension was determined by a tracking algorithm of fast Fourier transform-based correlation techniques. The shrinking or pulling of a single DNA chain can be monitored by measuring the DNA extension in time, while lowering or increasing the tethering force by moving the magnet slowly. Before measurements, the BSA in buffer has to be removed by rinsing with 1 mL PBS to avoid the interference of BSA in the process of DNA compaction. After isolating a single suspending lambda-DNA, the bead was pulled to its maximal displacement to the sidewall. Then, magnesium chloride at different concentrations and pH values were flushed to the flow cell and incubated for 10 min. The elastic response of DNA as a function a time was recorded and analyzed at different forces. The speed for the inflow of the DNA constructs or the counterion solution into the cell is 10 μL min^−1^. The force is determined by *F* = *k_B_T*<*L*>/*<δ*$x^{2}$*>*, where *k_B_* is Boltzmann constant, *T* is temperature, and <*L*> is average extension of single molecules, and *<δ*$x^{2}$*>* is the mean square displacement of the bead in the direction perpendicular to the applied force. A video camera was used to monitor the image of the structure in the focal plane, and it was used to record the position of the microsphere in real-time. The analysis of the extension was determined by a tracking algorithm by fast Fourier transform-based correlation techniques. The condensing force (Fc) is the force when the first step-like shrinking in the DNA extension-time curve occurred. When DNA is compacted, the magnetic bead is close to the sidewall to form a compact structure. It can be unraveled by moving the magnet toward the sidewall. The pulling force disassembling the DNA condensate is defined as unraveling force (Fu).

## 3. Results and Discussion

### 3.1. Electrophoretic Mobility of DNA Measurement

**Figure 2:**
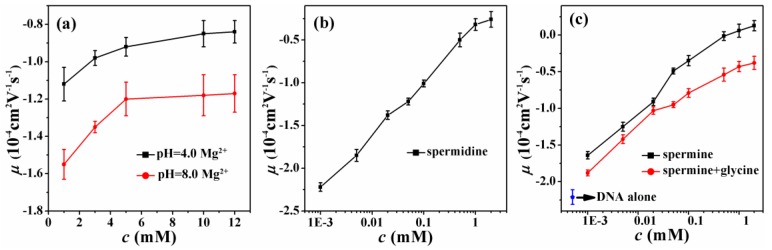
Electrophoretic mobility of DNA as a function of different ion concentrations. (**a**) Mg^2+^ (pH = 8.0, pH = 4.0). (**b**) spermidine. (**c**) spermine, spermine+2M glycine and DNA alone.

The electrophoretic mobility of a DNA complex depends linearly on its total charge, including the bare charge of DNA and the charge of counterions condensed on its surface. It reflects the extent of charge neutralization of DNA by counterions in solution. Figure 2 shows the electrophoretic mobility of DNA at various ionic conditions and different pH values to find out the critical charge neutralization for DNA compaction. Figure 2a shows the measured mobility of DNA as a function of Mg^2+^ concentration in solutions at pH = 8.0 and 4.0, respectively. We can see that the DNA mobility goes up positively with increasing concentrations of Mg^2^^+^ in solution. It is also notable that the promotion is almost saturated at the concentration of 10 mM Mg^2+^, although the mobility corresponding to the higher concentration of Mg^2+^ (12 nM) is also presented in the figure. Meanwhile, lowering pH in solution can increase DNA electrophoretic mobility significantly in the whole range of concentration of Mg^2+^. Figure 2b shows the measured mobility of condensed DNA as a function of the concentration of spermidine. It is notable that the mobility of DNA approaches zero, but is still negative at high concentrations of spermidine. Figure 2c shows the measured mobility of DNA as a function of spermine concentration and by introducing 2M glycine in solution. We can see that the DNA electrophoresis mobility changes from a negative to a positive value when the concentration of spermine is >0.5 mM, implying DNA charge inversion. The mobility of DNA alone is presented in the lower left corner in Figure 2c as the negative control experiment in the measurement. It is also notable that the mobility of DNA is suppressed after adding glycine.

**Figure 3:**
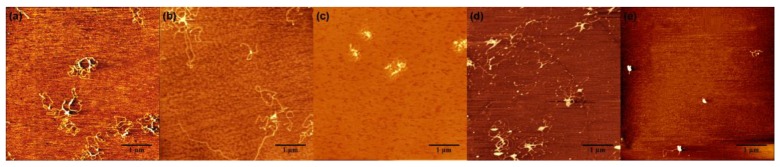
Atomic force microscopy (AFM) images of DNA forms induced by different ions at different solution conditions. (**a**) 3 mM Mg^2+^, pH = 4.0. (**b**) 100 μM spermidine. (**c**) 20 μM spermine (**d**) 50 μM spermine + 2 M glycine. (**e**) 50 μM spermine.

In order to find out the critical neutralization of DNA when its compaction starts, we imaged DNA morphology by AFM in different solutions containing divalent, trivalent, and quadrivalent ions. Figure 3 shows the starting stage of DNA compaction, where a condensing core appears and extensible threads of DNA surround the new-born core. Divalent Mg^2+^ does not induce DNA condensation at a normal pH value. But when the pH value decreases, we can see that DNA condensation occurs. Figure 3a shows AFM images of DNA morphologies at 3 mM Mg^2+^, pH = 4.0. We can see the flower morphologies of DNA condensation. We define the flower-like conformation as the starting stage of DNA compaction, where a condensing core appears and extensible threads of DNA surround the new-born core. The higher white dots in AFM images are the corns of the flower-like conformation. These are the occurrence of the critical condensation. The corresponding critical electrophoretic mobility of DNA condensation occurrence induced by divalent ions is about −0.98 × 10^−4^ cm^2^ V^−1^ s^−1^, as seen in Figure 2a. From the Figure 3b,c, DNA condensation occurrence induced by spermidine, spermine at the concentration 100 μM and 20 μM. However, the EM of DNA is also about −1.01 × 10^−4^ cm^2^ V^−1^ s^−1^, −0.91 × 10^−4^ cm^2^ V^−1^ s^−1^. Figure 3d shows the morphologies of DNA condensation at 50 μM spermine and 2M glycine solution. The morphologies are similar to the images in Figure 3a–c. We determined that the flower-like conformation is critical DNA compaction, where a condensing core appears and extensible threads of DNA surround the core. These are obviously different from free DNA and final state of globule morphologies. These results are consistent with divalent Mg^2+^. The electrophoretic mobility of DNA critical condensation is about −1 × 10^−4^ cm^2^ V^−1^ s^−1^.

It is strongly suggested that the main physical driving force of charge neutralization is electrostatic interaction, since we could suppress charge inversion of DNA in the same ion environment by increasing the dielectric constant of medium. We added glycine to the aqueous solution so that the dielectric constant of the medium has an added increment, depending on the concentration of these amino-carboxylic acids. When 2M glycine is added to the DNA-spermine solution at 50μM spermine concentration, the electrophoretic mobility changes from −0.49 × 10^−4^ cm^2^ V^−1^ s^−1^ to −0.95 × 10^−4^ cm^2^ V^−1^ s^−1^, implying a significant suppression of mobility or charge neutralization. Because of the increasing Coulombic repulsion, the condensed DNA by spermine can become less compact by adding glycine, as shown Figure 3d, in which the compact DNA globules in Figure 3e become loose condensed structures with some DNA threads visible by adding glycine in preparation.

In our previous study, the ratio of the saturated electrophoretic mobility of DNA in monovalent and divalent cation solution is about 1:2, and the ratio becomes 1:4.5 for the case of monovalent versus trivalent counterions [28,29,30]. In the framework of the Manning theory, we can infer the critical charge neutralization of DNA for its compaction. By simple linear interpolation, we can calculate that the corresponding charge neutralization fraction is about 89% for −1.0 × 10^−4^ cm^2^ V^−1^ s^−1^ when 88% charge fraction for divalent counterions is −1.2 × 10^−4^ cm^2^ V^−1^ s^−1^, and 92% charge fraction for trivalent counterions corresponds to the electrophoretic mobility of −0.4 × 10^−4^cm^2^V^−1^s^−1^. This small difference of charge neutralization is very important for the regulation of DNA condensation and decondensation in processes such as DNA transcription. 

### 3.2. AFM Morphology of DNA

**Figure 4:**
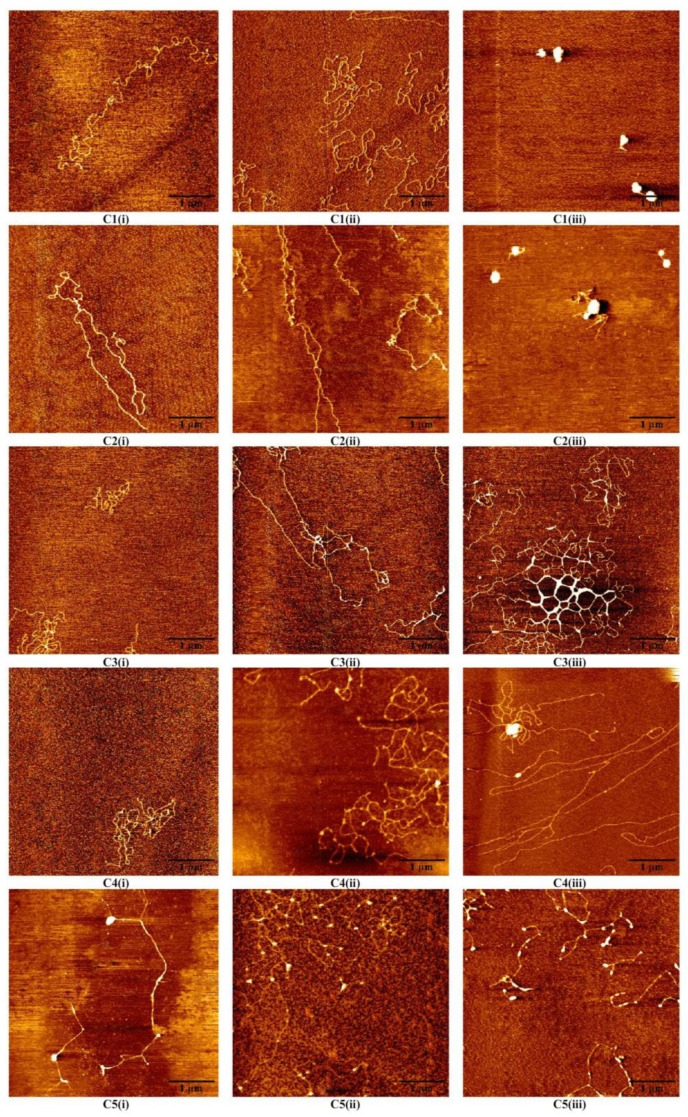
AFM images of DNA forms induced by different concentrations of Mg^2+^ and pH values (pH = 8.0, 5.0, 3.0). C1(i)–C5(i) pH = 8.0, C1(ii)–C5(ii) pH = 5.0 C1(iii)–C5(iii) pH = 3.0; C1(i)–C1(iii) 3 mM Mg^2+^. C2(i)–C2(iii) 10 mM Mg^2+^. C3(i)–C3(iii) 20 mM Mg^2+^. C4(i)–C4(iii) 50 mM Mg^2+^. C5(i)–C5(iii) 100 mM Mg^2+^.

**Figure 5:**
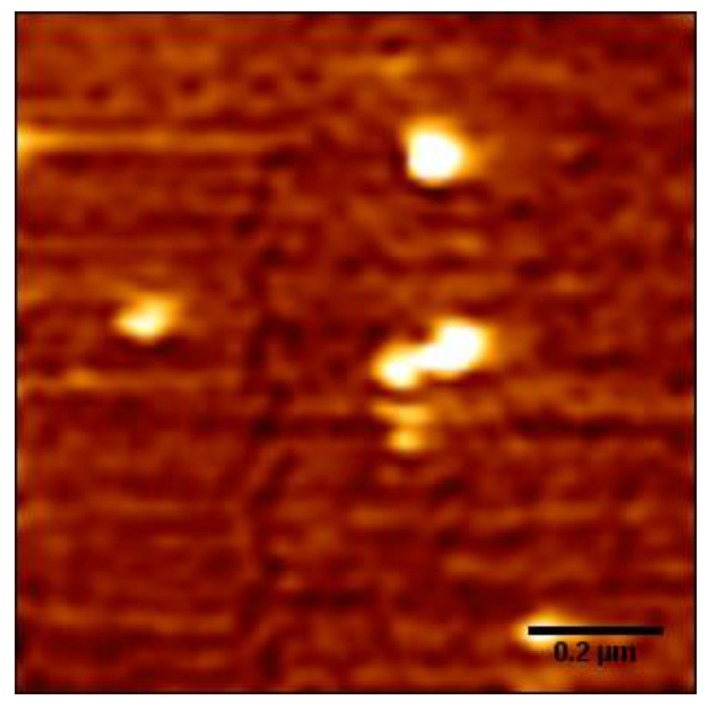
AFM image of DNA in 3 mM Mg^2+^ at pH = 3.0 in liquid.

Figure 4(C1)–(C5) shows AFM images of DNA morphologies at different pH values (pH = 8.0, 5.0, 3.0) at five different Mg^2+^ concentrations of 3 mM (Series 1 C1), 10 mM (Series 2 C2), 20 mM (Series 3 C3), 50 mM (Series 4 C4), 100 mM (Series 5 C5). Figure 4(C1, i–iii) shows that the DNA morphologies change from random coil to globular state at 3 mM Mg^2+^, when the pH in solution goes down from 8.0 to 3.0. Mg^2+^ at pH = 3.0 is able to induce DNA to a stable globular state, while DNA morphologies at pH = 5.0 are still at random coil states, as can be seen. Thus, the DNA condensation occurs at a pH lower than 5.0. Figure 4(C2, i–iii) shows that at 10 mM Mg^2+^ and at different pH values, the DNA conformation is similar to the results at 3 mM Mg^2+^. This means that the morphologies at pH 8.0 and 5.0 are freely extensive and the globule state occurs at pH 3.0. When the concentrations of Mg^2+^ change from 20 mM to 100 mM in Figure 4(C3)–(C5), the DNA conformation changes slight. Figure 4(C1, iii)–(C5, iii) shows that the globular conformation will decompact to loose coil and flower structures. The decondensation induced by a higher concentration of Mg^2+^ occurs. This may be the intermediate state between loose patterns and globular patterns. The morphologies at pH = 8.0 and 5.0 are similar, but condensation occurs clearly at pH = 3.0. The effective pH range of tris buffer is between 7.5 and 9.0. Indeed, tris has no buffer function when the pH value of solution is lower than 6.0. In our experiment, we want to keep the same solution conditions, to obtain consistent results. At the increase of tris concentration, mobility would become smaller, which has mentioned in Bestman’s research [5]. As we have discussed before, DNA morphologies by AFM in liquid might be slightly different from those in air, but the difference is not important, as the latter is much easier for sample preparation and better quality of imaging in practice [27]. For consistency, we observe the DNA morphologies in 3 mM Mg^2+^ at pH = 3.0 in liquid by AFM, as presented it in Figure 5. We can see that the morphologies of DNA are similar to those in air as in Figure 4(C1, iii), but with lower resolutions. Actually, the AFM images show a flattened morphology of DNA condensates, but keep their original compacting structures, which has also been confirmed by Balhorn and his colleagues [26].

### 3.3. The Condensing Forces and the Unraveling Forces Measurement

**Figure 6:**
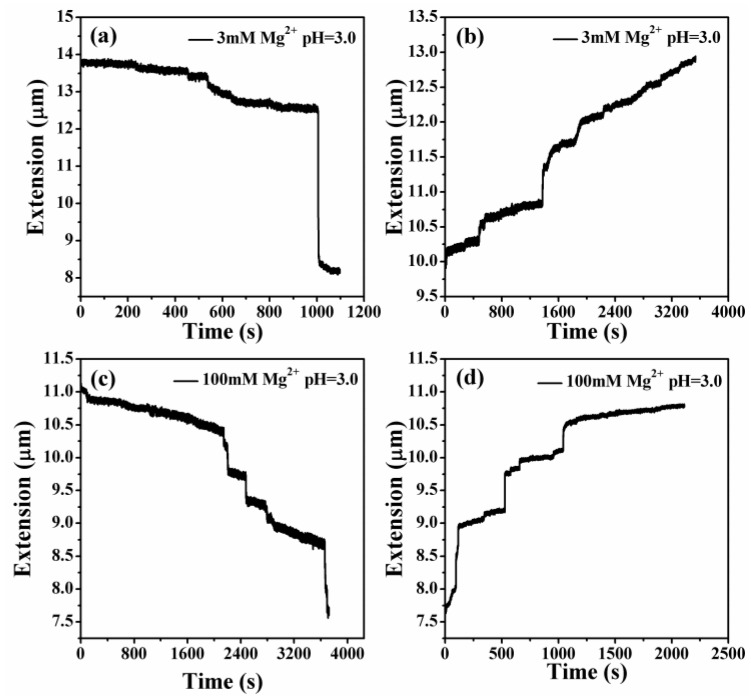
DNA extension-time curve measured by MT in DNA, the releasing and stretching process at different concentration of Mg^2+^ in 10mM tris. (**a**), (**b**) 3 mM Mg^2+^, pH = 3.0. (**c**), (**d**) 100 mM Mg^2+^, pH = 3.0.

In MT measurement, we first have to find a single tethered λ-DNA molecule, whose extension is close to 16 μm under high tension (>10 pN) in PBS. Then, we flow different concentrations of Mg^2+^ at different pHs to the sample cell and apply force to the DNA by moving the magnets slowly. In the contracting and pulling process of DNA, the condensing and unraveling forces are measured, respectively. Figure 6 shows the process of DNA contraction and unraveling in a solution of 3 mM Mg^2+^ and 100 mM Mg^2+^ at pH 3.0. When the pH of solution is changed to about 3.0, the extension-time curve appears to show the normal discontinuous stepwise contraction, or unraveling, as shown in Figure 6a,b.

**Figure 7:**
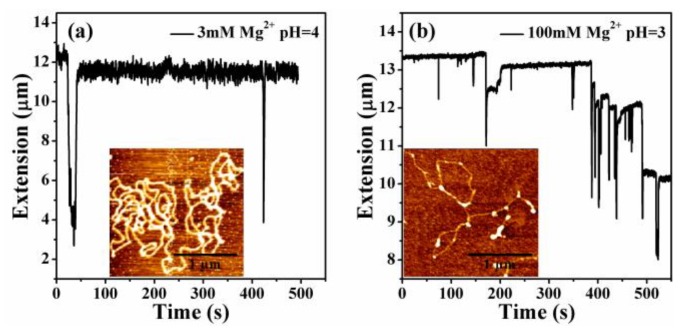
DNA extension-time curve measured by MT in DNA, the releasing and stretching processes at different concentrations of Mg^2+^ and pH value. (**a**) 3 mM Mg^2+^, pH = 4.0. (**b**) 100 mM Mg^2+^, pH = 3.0.

It can be seen that the condensation morphologies at lower concentrations and pH values are at the final globular state. When increasing the pH value and the concentration of divalent ions, DNA is in an unstable transition state, being able to switch between compact and extensible states. We can see that the extension-time curve shows the large stepwise and unstable jump up and down processes, corresponding to an unstable intermediate state. Figure 7a,b shows DNA extension-time curves measured by MT in DNA releasing processes at 3 mM Mg^2+^ in 10 mM tris-HCl, pH = 4.0 and 100 mM Mg^2+^ in 10 mM tris-HCl, pH = 3.0. Thus, we found that DNA compaction by divalent cations at low pH is weakened and even decondensed with increasing concentrations of counterions. The details of DNA stretching and releasing by MT in Figure 6 and Figure 7 can be found in the raw data included as Supplementary Information (S1).

From the DNA extension-time curve on the release and stretching processes, the extension of DNA changes with time. It is discontinuous and stepwise, and the extension of DNA on the final stretching process cannot be stretched by the contour length when the force is above 10 pN. This may be because the persistence length of DNA changed, accompanied by DNA condensation. To quantify this observation, we measured the condensing and unraveling forces of DNA complexes. Table 1 lists Fc and Fu of DNA in solution of various concentrations of Mg^2+^ at different pH values. We can see that the DNA condensation increased with the increasing of the concentration of Mg^2+^. When the concentrations are increased to 100 mM, the Fc changes a smaller amount than the value at 10 mM Mg^2+^. The Fu of DNA complexes shows the same tendency as the condensing force. However, the force increased with the decrease in pH value. We repeat all measurements at least 3 times to obtain consistent results, and the standard deviation is calculated as the error accordingly.

## 4. Discussion

**Figure 8:**
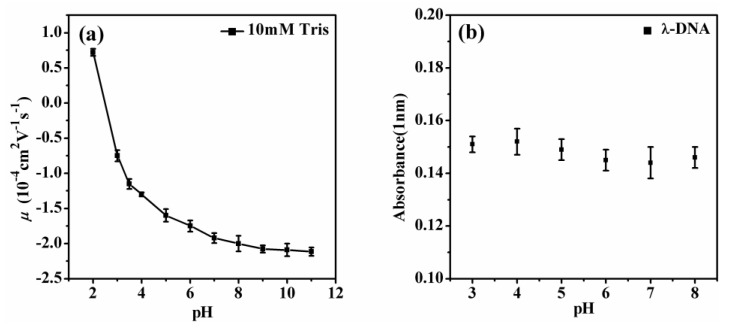
(**a**) Electrophoretic mobility of DNA as a function of different pH values. (**b**) The absorbance of DNA at 260 nm at pH value from 3.0 to 8.0.

In our previous work, we focused on the pH effects on DNA compaction in tri- and tetravalent cations, and how it enables the condensation of DNA at physiological conditions [31]. The present study focuses on the charge neutralization and compaction of DNA at very low pH, which may occur in the domain of DNA nanotechnology, but is apparently out of the range of physiological conditions of cells [32]. When the pH of solution decreases, DNA may cross its isoelectric point and undergo melting of its double helix structure, due to the multiple charge groups and hydrogen bonds in DNA [33]. We measured the electrophoretic mobility of DNA at pH from 2.0 to 11.0, and presented them in Figure 8a. We found that the sign of electrophoretic mobility went from negative to positive when the pH changed from 3 to 2, implying that phosphate groups are dominant in this measurement [34]. Therefore, we have known that DNA might melt to some extent under strong acidic conditions, depending on the valence and concentration of cations in a solution [31]. To clarify the influence of DNA melting on charge neutralization and compaction of DNA, we measured the melting degree of DNA in the same experimental conditions by hypochromic effect. The absorbance of DNA at 260 nm can increase about 50% when the double helix structure of the DNA is fully melted to two single strands of DNA. The measurement was achieved by the micro-volume Quawell Q5000 UV-Vis spectrophotometer (San Jose, CA, USA) and the result is presented in Figure 8b. We can see that the absorbance of DNA at 260 nm increases less than 5% when pH goes down from 8.0 to 3.0. Thus, the melting of DNA is less than 10% by linear extrapolation, implying that only a small fraction of DNA is melted and most of its double-stranded structure is maintained. Therefore, our conclusion about DNA charge neutralization and compaction by di- to tetra-valent cations under strong acidic conditions is still approximately valid, although DNA melting might result in some small modifications.

**Figure 9:**
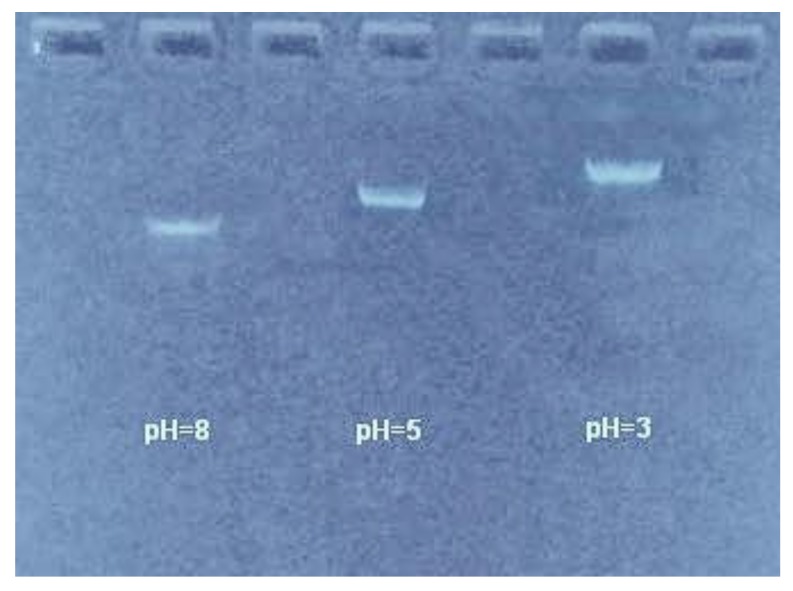
The agarose gel electrophoresis of DNA in the presence of 3mM Mg^2+^^.^at different pH values (8.0, 5.0, 3.0).

In order to further confirm DNA condensation by divalent cations at low pH, we ran agarose gel electrophoresis of DNA at different pH values (8.0, 5.0, 3.0) in 3 mM Mg^2+^ solution, and the result is shown in Figure 9. We can see that DNA electrophoretic mobility becomes significantly slower when the pH of a solution is decreased, implying that DNA condensation occurs in the case that was observed by AFM.

However, divalent cations are unable to condense DNA under mild pH conditions. In our present study, we directly demonstrated DNA compaction and its regulation as mediated by divalent cations by lowering the pH of a solution. At the same time, we found that DNA compaction by divalent cations at low pH is weakened and even decondensed when the concentration of counterions is increased. Mg^2+^ resides in the negatively-charged pockets formed by the phosphate groups of the DNA backbone in a compaction state, resulting in DNA interlocked in the minor-grove-to-minor-grove conformation [35]. When lowering the pH of a solution, the interlocks are strengthened to such an extent this enables us to tether the compact DNA chain, because of the decrease in the average fractional ionization of the polyelectrolyte chain. Low-pH-induced DNA structural changes in the presence of low concentrations of Mg^2+^ were investigated in the pH range 6.8–2.1 by Raman microspectroscopy. These are due to protonation and unstacking of the DNA bases during DNA melting, and also to changes in the DNA backbone conformation [36]. The confocal Raman microspectrometer is used to investigate the influence of Na^+^ and Mg^2+^ ions on the DNA structural changes induced by low pH. The concentrations of Mg^2+^ can affect the degree of protonation. Mg^2+^ is found to be more effective in protecting DNA against binding of H^+^ when compared to Ca^2+^, as presented in a previous study. Divalent metal cations (Mg^2+^, Ca^2+^) are more effective in protecting DNA against protonation than monovalent ions (Na^+^) [37]. In our experiment, lower pH values can increase the protonation of DNA. At a pH 3.0 and 2.0, condensed globular aggregates of the Mg-DNA complexes were observed at small concentrations of Mg^2+^. In general, ions of Mg^2+^ reside in the negatively charged pockets formed by phosphate groups of the DNA backbone in compaction states, resulting in DNA interlocks in the minor-grove-to-minor-grove conformation. When lowering the pH of a solution, the interlocks are strengthened to such an extent they enable us to tether the compact DNA chain, because of the decrease in average fractional ionization of the polyelectrolyte chain. However, high concentrations of Mg^2+^ might prevent protonation of guanine, cytosine, and adenine in DNA, leading to DNA decompaction to some extent.

**Figure 10:**
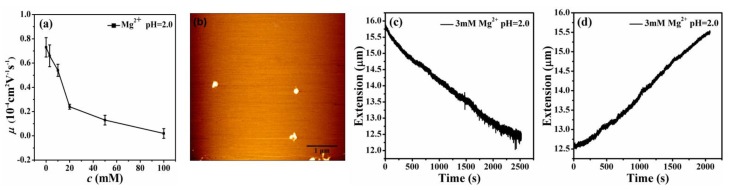
(**a**) Electrophoretic mobility of DNA as a function of the concentration of divalent ions at pH = 2.0. (**b**) AFM images of DNA forms induced by 3 mM Mg^2+^ at pH = 2.0. (**c**,**d**) DNA extension-time curves measured by MT in DNA, the releasing and stretching processes at 3 mM Mg^2+^, pH = 2.0.

Figure 10a shows the measured mobility of condensed DNA as a function of different concentrations of Mg^2+^ at pH = 2.0. In general, we can see that the mobility of DNA shifts to a less negative value after decreasing the pH value. But when the pH value changes to 2.0, the mobility of DNA goes up from negative to positive values. The mobility of DNA at pH 3.0 is almost negative and the mobility of DNA at pH 2.0 is almost positive. Figure 10b shows that at 3mM Mg^2+^ and at pH = 2.0, the DNA morphologies change to globular state. The condensation is clear. It is notable that the imaging was accomplished for the adsorbed DNA molecules on a mica disk in air, not in solution. In this case, AFM images show the flattening morphology of DNA condensates, but keep their original compacting structures, which is similar to those in solution, but with smaller heights (the axial direction). Figure 10c,d shows the results of MT at pH = 2.0. When the pH value is 2.0, we can see that the extension of DNA changes almost linearly with time, even when a larger pulling force (>10 pN) is applied, in which an apparent DNA condensation occurred in divalent counterion solution.

It is notable that the critical charge neutralization of DNA for compaction is only 1% or even less (if including the nonlinear effect of charge screening), which is higher than the saturated charge fraction of DNA in a divalent ion solution. Thus, divalent ion-mediated like-charge attraction by pH regulation is quite sensitive.

## 5. Conclusions

In summary, we found that the critical DNA charge neutralization needed for DNA attraction or compaction is about 89% by analyzing DNA compaction in tri- and tetravalent counterions ionic conditions. At the critical point, the corresponding electrophoretic mobility of DNA is promoted to around −1.0 × 10^−4^ cm^2^ V^−1^ s^−1^. It is also valid for DNA compaction by divalent counterions in a low pH solution. In the process of pH and ionic regulation, we found that DNA compaction by divalent cations at low pH (pH < 5.0) is weakened and even decondensed with an increasing concentration of counterions. For example, the stable compaction of DNA at pH = 3.0 enters an unstable transition state when the concentration of Mg^2+^ goes up from 3 mM to 100 mM. In addition, when the pH is extremely low (such as below 2.0), the extension of DNA changes almost linearly, but no stepwise jumps with time even when a large pulling force (>10 pN) are applied. It is notable that the present conclusion is only valid in vitro, and out of the range of physiological conditions, since DNA may partly denature in extreme conditions.

## Figures and Tables

**Table 1:** The condensing forces (Fc) and the unraveling forces (Fu) of DNA complexes as the concentration of magnesium chloride and pH value.

pH Value	Fc (pN)			Fu (pN)		
	3 mM	10 mM	100 mM	3 mM	10 mM	100 mM
5.0	0.0±0.0	1.1±0.2	0.0±0.0	0.0±0.0	0.8±0.3	0.0±0.0
3.0	2.4±0.6	3.8±0.8	2.6±0.8	3.7±0.8	7.6±0.5	6.2±0.7
2.0	3.4±1.2	4.5±0.9	3.1±1.0	7.2±1.5	9.5±1.4	5.8±0.9

## Supplementary Materials

The following are available online at http://www.mdpi.com/2073-4360/11/2/337/s1, Figure S1. DNA extension-time curve measured by MT in DNA releasing and stretching process at different concentration of Mg^2+^ in 10 mM Tris. (a,b) 3 mM Mg^2+^, pH = 3.0. (c,d) 100 mM Mg*2+*, pH = 3.0. Figure S2. DNA extension-time curve measured by MT in DNA releasing and stretching process at different concentration of Mg^2+^ and pH value. (a) 3 mM Mg^2+^, pH = 4.0. (b) 100 mM Mg^2+^, pH = 3.0.
