# Supplementary material for: DNA Compaction and Charge Neutralization Regulated by Divalent Ions in very Low pH Solution
Source: Polymers (Basel). 2019 Feb 15;11(2):337. doi: 10.3390/polym11020337 (PMC6419228; doi:10.3390/polym11020337)
Supplement: Supplementary file 1 [file polymers-11-00337-s001.pdf]

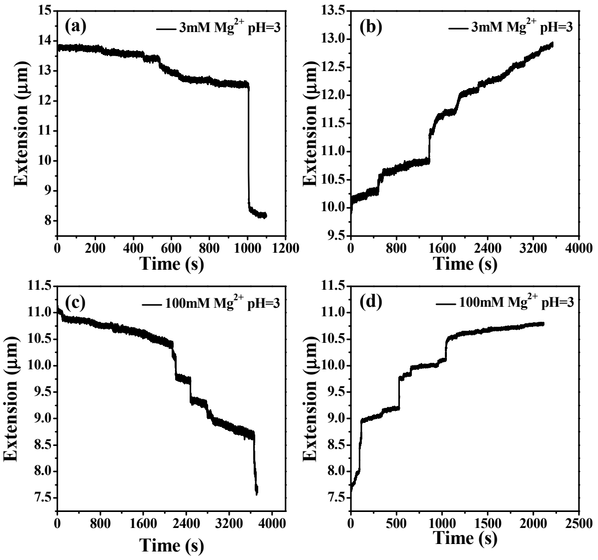

Figure S1. DNA extension-time curve measured by MT in DNA releasing and stretching process at different concentration of Mg^{2+} in 10mM Tris. (a),(b):3mM Mg^{2+},pH=3.0. (c),(d):100mM Mg^{2+}, pH=3.0.

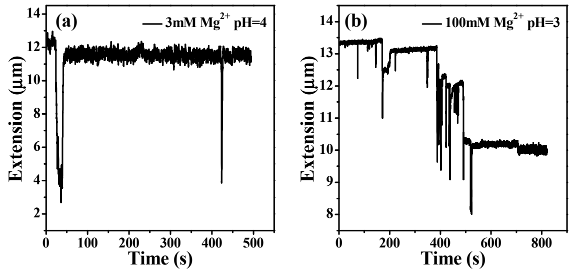

Figure S2. DNA extension-time curve measured by MT in DNA releasing and stretching process at different concentration of Mg^{2+} and pH value.(a):3mM Mg^{2+}, pH=4.0.(b):100mM Mg^{2+},pH=3.0.
